# Calcium sensing receptor protects high glucose-induced energy metabolism disorder via blocking gp78-ubiquitin proteasome pathway

**DOI:** 10.1038/cddis.2017.193

**Published:** 2017-05-18

**Authors:** Yuehong Wang, Ping Gao, Can Wei, Hongzhu Li, Li Zhang, Yajun Zhao, Bo Wu, Ye Tian, Weihua Zhang, Lingyun Wu, Rui Wang, Changqing Xu

**Affiliations:** 1 Department of Pathophysiology, Harbin Medical University, Harbin 150086, China; 2 The Key Laboratory of Cardiovascular Medicine Research (Harbin Medical University), Ministry of Education, Harbin 150086, China; 3 Department of Endocrinology, The Second Affiliated Hospital of Harbin Medical University, Harbin 150086, China; 4 The Cardiovascular and Metabolic Research Unit, Laurentian University, Sudbury, Ontario, Canada P3E 2C6; 5 Health Sciences North Research Institute, Sudbury, Ontario, Canada P3E 5J1

## Abstract

Diabetic cardiomyopathy (DCM) is a major complication and fatal cause of the patients with diabetes. The calcium sensing receptor (CaSR) is a G protein-coupled receptor, which is involved in maintaining calcium homeostasis, regulating cell proliferation and apoptosis, and so on. In our previous study, we found that CaSR expression, intracellular calcium levels and cardiac function were all significantly decreased in DCM rats; however, the exact mechanism are not clear yet. The present study revealed the protective role of CaSR in myocardial energy metabolism disorder induced by high glucose (HG) as well as the underlying mechanism. Here, we demonstrated that HG decreased the expression of CaSR, mitochondrial fusion proteins (Mfn1, Mfn2), cell gap junction related proteins (Cx43, *β*-catenin, N-cadherin), and intracellular ATP concentration. In contrast, HG increased extracellular ATP concentration, the expression of gp78, mitochondrial fission proteins (Fis1, Drp1), and the ubiquitination levels of Mfn1, Mfn2 and Cx43. Moreover, CaSR agonist and gp78-siRNA significantly reduced the above changes. Taken together, these results suggest that HG induces myocardial energy metabolism disorder via decrease of CaSR expression, and activation of gp78-ubiquitin proteasome system. In turn, these effects disrupt the structure and function of the mitochondria and the cell gap junction, result in the reduced ATP synthesis and the increased ATP leakage. Stimulation of CaSR significantly attenuates HG-induced abnormal myocardial energy metabolism, suggesting CaSR would be a promising potential therapeutic target for DCM.

Myocardial diastolic and systolic dysfunction is main characteristic of diabetic cardiomyopathy (DCM),^1^ which is mainly related to abnormal synthesis and utilization of ATP.^2^ The abnormal myocardial energy metabolism will inevitably impair myocardial structure and function, eventually increasing the risk of myocardial infarction and heart failure.^3^

Mitochondria are well established as the organelles responsible for cellular energy production, such that cellular ATP production is dependent on normal mitochondrial structure and function. Mitochondrial fusion-related proteins (Mfn1, Mfn2) and mitochondrial fission-related proteins (Fis1, Drp1) maintain the dynamic balance of mitochondrial structure together.^4, 5^ Cardiac energy demand is very large, requiring myocardial cell to produce more ATP by the fusion of the mitochondria.^6, 7^ Obviously, the decreased expression of Mfn1 and Mfn2 will result in the decrease of ATP synthesis. So, elucidating the regulatory mechanisms of Mfn1 and Mfn2 expression is critical to understanding and treating DCM.

The ubiquitin proteasome pathway is the predominant protein degradation pathway,^8^ and its activation is regulated by various proteins, such as gp78.^9^ This protein also forms a specialized tubule between the closely adjacent endoplasmic reticulum and mitochondria, and facilitates the rapid transfer of calcium from the former to the latter.^10^

Besides, gap junction intercellular communication (GJIC) consists of hemichannel, which can quickly transmit the materials, energy and electrical signals between adjacent cells.^11, 12^ Impaired GJIC will result in the leakage of intracellular ATP. Therefore, the abnormal energy metabolism is not only related to the decrease of ATP synthesis, but also to the increase of ATP leakage.

It is reported that DCM can cause the decrease expression of mitochondrial fusion protein and gap junction protein,^13, 14^ although the mechanisms underlying these effects are not established. In addition, whether the activation of the gp78-ubiquitin proteasome system is involved in DCM is not clear.

Calcium sensing receptor (CaSR) is a G protein-coupled receptors that is widely expressed in prokaryotic and eukaryotic cells. Early study found that the function of CaSR is to regulate the release of parathyroid hormone and maintain the body's calcium homeostasis.^15^ Furthermore, CaSR is also closely related to cell differentiation, proliferation, migration, apoptosis, brain development, wound healing, and so on.^16, 17, 18^ The increase of extracellular calcium concentration can directly activate CaSR, and some amino acids are also involved in the regulation of CaSR activation.^19^ Our previous study found that CaSR is involved in myocardial ischemia reperfusion injury, myocardial infarction and pulmonary hypertension, and so on.^20, 21, 22^ And we recently observed that the cardiac function and the expression of CaSR were decreased in the rat diabetic myocardium.^23^ Nevertheless, the mechanism underlying the observed association between the CaSR and ATP remains unclear.

In present study, by using high glucose (HG)-induced energy metabolism disorder model in the primary cultured cardiomyocytes, we studied the effect of CaSR in maintaining ATP content and protecting the structure and function of mitochondria and gap junction. The underlying mechanisms were also explored.

## Results

### Downregulation of CaSR expression is induced by HG in primary neonatal rat cardiomyocytes

**Figure 1:**
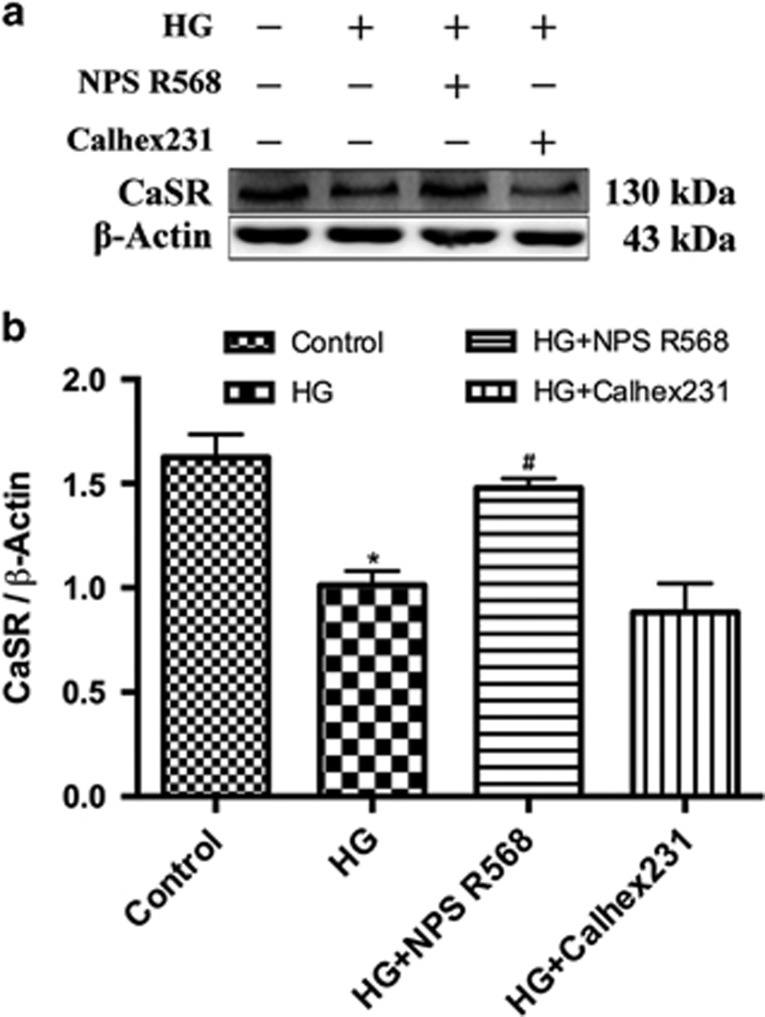
The effect of HG on CaSR expression in primary cultured neonatal rat cardiomyocytes. The cardiomyocytes cultured in Control group (5.5 mM) and HG group (40 mM) with or without 5 *μ*M NPS R568 or 3 *μ*M Calhex231 for 48 h were collected for immunoblotting analysis of CaSR expression. (**a**) Representative western blot of CaSR expression in cardiomyocytes exposed to HG in the presence of 5 *μ*M NPS R568 or 3* μ*M Calhex231; (**b**) The CaSR protein levels normalized by *β*-actin. All data expressed as means±S.E. (*n*=3). *P<0.05 *versus* Control group; ^#^*P*<0.05 *versus* HG group

We detected the expression of CaSR in primary neonatal rat cardiomyocytes by immunoblotting with *β*-actin as a reference. The results showed that compared with the control group, the expression of CaSR in HG group was significantly decreased. Furthermore, the expression of CaSR in HG group was increased or decreased by the pretreatment of CaSR agonist (NPS R568) or CaSR antagonist (Calhex231), respectively (Figures 1a and b).

### HG damages mitochondrial structure and function by downregulating expression of CaSR

**Figure 2:**
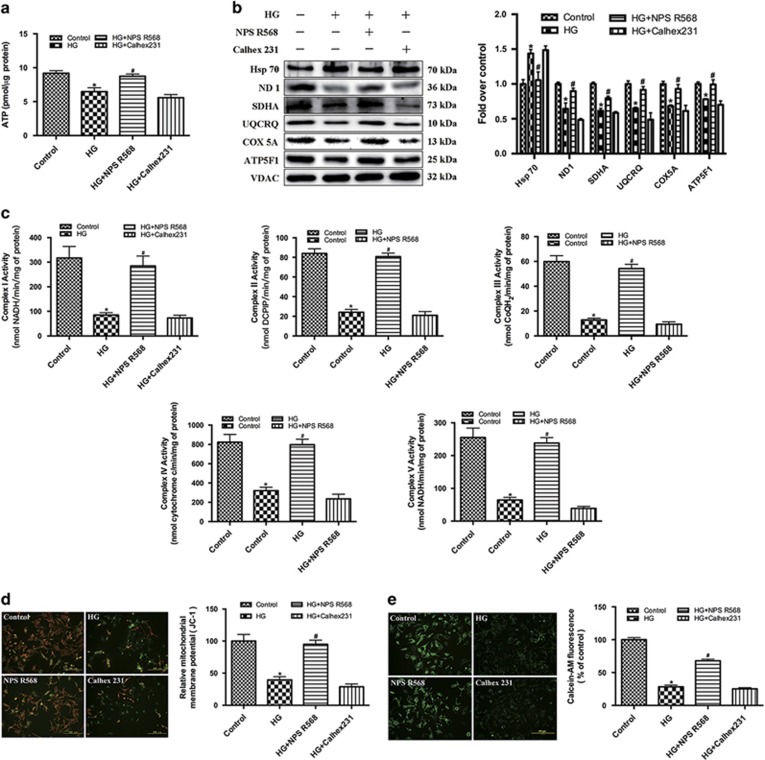
Mitochondrial dysfunction caused by HG was attenuated through CaSR activation. The cardiomyocytes were cultured in control group (5.5 mM) and HG group (40 mM) with or without 5 *μ*M NPS R568 or 3 *μ*M Calhex231 for 48 h. (**a**) The intracellular ATP of each group was detected by chemiluminescence (*n*=3). (**b**) Representative western blot of UQCRQ, ND1, ATP5F1, COX5A, SDHA and Hsp70 in comparison with VDAC expression in cardiomyocytes exposed to HG in the presence of 5 *μ*M NPS R568 or 3 *μ*M Calhex231 (*n*=3). (**c**) Activities of complex (*I*–*V*) were detected by UV Spectrophotometry (*n*=4). (**d**) Δ*ψ*m was measured by JC-1 staining and the images were obtained by fluorescent microscopy. Scale bars=200 *μ*m. The average fluorescence intensities are expressed as the ratio of red to green (*n*=10). (**e**) Calcein-AM was used to measure the changes of mPTP opening. Cells in different groups were stained by the calcein-AM. The images were obtained by fluorescent microscopy. Scale bars=200 *μ*m (*n*=10). The changes in fluorescence intensity are varies inversely with the opening degree of mPTP. *P<0.05 *versus* control; ^#^*P*<0.05 *versus* HG

The results of chemiluminescence showed that the content of intracellular ATP in HG group was significantly lower than in control group. NPS R568 or Calhex231 pretreatment could significantly reduce or enhance the changes above (Figure 2a).

Heat shock protein 70 (Hsp70) is generally regarded as a marker of stress response. We firstly detected Hsp70 expression by immunoblotting with VDAC as a reference. The results showed that the expression of Hsp70 was significantly upregulated in HG group (Figure 2b), indicating that HG induces the stress response.

The proton-transferring ability of mitochondrial respiratory chain is crucial to produce ATP.^24^ We observed that the expression of mitochondrial respiratory chain complex (*I*–*V*) related proteins (ND1, SDHA, UQCRQ, COX5A, ATP5F1) and the activities of mitochondrial respiratory chain complex (*I*–*V*) were decreased in HG group. NPS R568 or Calhex231 pretreatment could weak or enhance the above-mentioned effect induced by HG (Figures 2b and c).

Calcein-AM cobalt technology was used to detect the status (i.e., open *versus* closed) of the mitochondrial permeability transition pore (mPTP).^25^ The fluorescence intensity in the control group was set as 100%, the fluorescence intensity in the HG group was significantly lowered to 28.34±2.49%. NPS R568 increased the fluorescence intensity from 28.34±2.49% to 67.95±2.36%, while Calhex231 pretreatment further decreased the fluorescence intensity from 28.34±2.49% to 25.25±1.72% (Figure 2d). An open mPTP has been previously shown to decrease the mitochondrial membrane potential (ΔΨm).^26^ In the present study, the red/green fluorescence ratio in the control group was set as 100%. The ratio was significantly decreased to 39.61±4.98% in HG group. NPS R568 or Calhex231 pretreatment can increase or decrease the ratios from 39.61±4.98% to 95.11±6.34% or 28.81±4.40% (Figure 2e).

**Figure 3:**
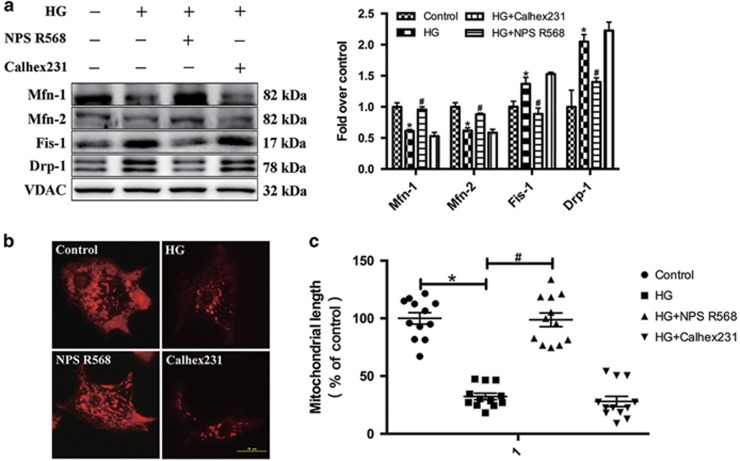
Effect of HG and CaSR activation on the expression of mitochondrial fusion and fission-related proteins. The inhibition of mitochondrial fusion by HG were attenuated by NPS R568. Expression of Mfn1, Mfn2, Fis1 and Drp1 in cultured cardiomyocytes in Control group (5.5 mM) and HG group (40 mM) after various treatment for 48 h. (**a**) Representative western blot of Mfn1, Mfn2, Fis1 and Drp1 in comparison with VDAC expression in cardiomyocytes exposed to HG in the presence of 5 *μ*M NPS R568 or 3 *μ*M Calhex231. (**b**) Morphology of mitochondria were detected by Mito-Tracker and photo were taken by fluorescence microscopy. Scale bars=10 *μ*m (*n*=12). (**c**) The average length of the mitochondria in each group was quantified (*n*=12). *P<0.05 *versus* control; ^#^*P*<0.05 *versus* HG

To elucidate the mechanism underlying the observed HG-induced mitochondrial respiratory chain disruption, immunoblotting was used to analyze the mitochondrial structure associated proteins *in vitro*. The results showed that the expression of mitochondrial fusion-related proteins (Mfn1, Mfn2) was decreased and the expression of mitochondrial fission-related proteins (Fis1, Drp1) was increased in HG group. NPS R568 attenuated the changes induced by HG, but no significant changes were observed after pretreatment with Calhex231 (Figure 3a).

The mitochondrial morphology was detected with the Mito-Tracker (red) staining.^27^ In the control group, the red dye was found to be widely distributed, and exhibited a ‘clubbed’ pattern, while in contrast, it was sparsely distributed, and assumed a ‘dotted’ pattern in HG group. (Figure 3b). NPS R568 or Calhex231 pretreatment can reduce or increase the changes caused by HG (Figure 3b). Then the mitochondrial length of the control group was standardized as 100%, the mitochondria length in the HG group was decreased to 32.37±2.79% (Figure 3c). NPS R568 or Calhex231 pretreatment can increase or decrease the mitochondria length from 32.37±2.79% to 98.75±5.95% or 28.02±4.41% (Figure 3c).

These data clearly indicate that HG can damage the function and structure of mitochondria in cardiomyocytes, decrease the synthesis of ATP. In contrast, activation of CaSR provides protective effect.

### The effect of HG and CaSR on gap junction

**Figure 4:**
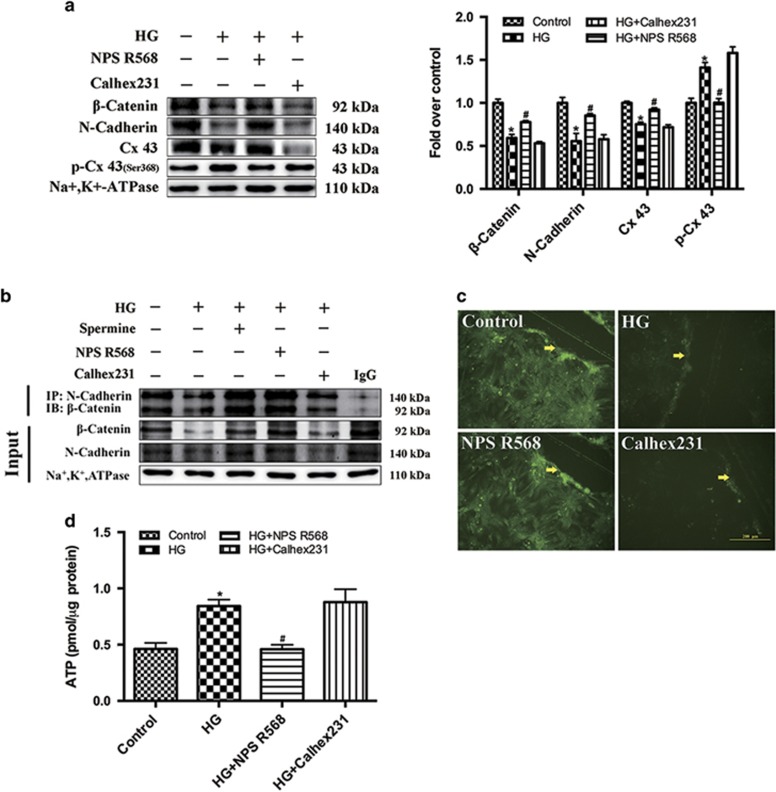
Activation of CaSR blocks HG-induced Cx43 degradation and stabilizes function of cell gap junction. HG-induced degradation of *β*-catenin, N-cadherin and Cx43. NPS R568 could decrease the effect of HG. (**a**) Representative western blot of *β*-catenin, N-cadherin and Cx43 in comparison with Na^+^, K^+^-ATPase expression in cardiomyocytes exposed to HG in the presence of 5 *μ*M NPS R568 or 3 *μ*M Calhex231 (*n*=3). (**b**) The expression of *β*-catenin and N-cadherin complex were detected (*n*=3). A typical blot is shown. (**c**) The function of Cx43 was measured by scrape-loading dye transfer technique (SLDT) and photoed by fluorescence microscopy. Scale bars=10 *μ*m (*n*=3). The yellow arrow represents the scratch. (**d**) The extracellular ATP content of each group was detected by chemiluminescence (*n*=3). *P<0.05 *versus* control; ^#^*P*<0.05 *versus* HG

We first detected the cell membrane associated gap junction protein by immunoblotting with Na^+^ K^+^-ATPase as a reference. The results showed that the expression level of *β*-catenin, N-cadherin and Cx43 were significantly decreased in the HG group, while the expression level of Cx43 phosphorylation at Ser368 was significantly upregulated in HG group. NPS R568 lightened the changes induced by HG, but no significant changes occurred after pretreatment with Calhex231 (Figure 4a).

Previous study have demonstrated that the downregulation of *β*-catenin and N-cadherin expression hinders both the complexes formation and cell adhesion, which then destroys GJIC ultimately.^28^ In the present study, the results of immunocoprecipitation showed that the expression level of complex was significantly decreased in the HG group. NPS R568 and Spermine (a non-specific agonist) pretreatment could significantly enhance the complex level in HG group, while Calhex231 pretreatment could reduce the complex level (Figure 4b).

**Figure 5:**
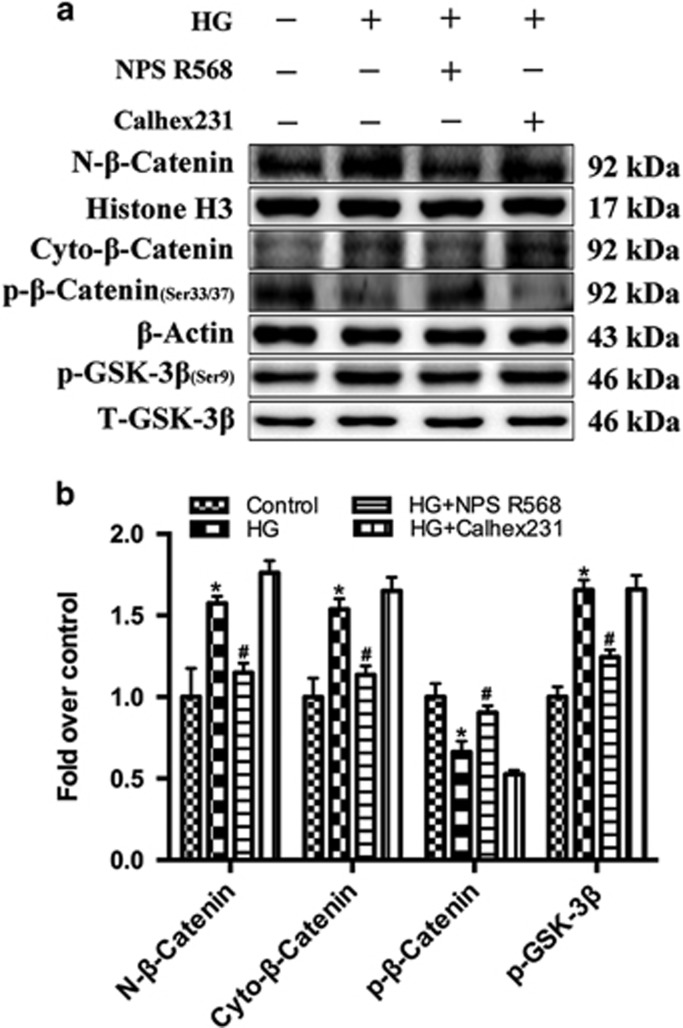
Activation of CaSR inhibits the phosphorylation of GSK-3*β* and the nuclear translocation of *β*-catenin induced by HG. HG promoted the nuclear translocation of *β*-catenin, which was attenuated by NPS R568 pretreatment. (**a**) The whole-cell proteins from primary neonatal rat cardiomyocytes in control group (5.5 mM) and HG group (40 mM) after various treatment conditions for 48 h were detected by immunoblotting with antibodies against N-*β*-catenin, Cyto-*β*-catenin, p-*β*-catenin, p-GSK-3*β* and total GSK-3*β*. A representative blot was shown. (**b**) The protein levels of N-*β*-catenin were quantified as a ratio against Histone H3. The expression of Cyto-*β*-catenin and p-*β*-catenin were quantified as a ratio against *β*-actin. The expression of p-GSK-3*β* were quantified as a ratio against total GSK-3*β* (*n*=3). *P<0.05 *versus* control; ^#^*P*<0.05 *versus* HG

The effect of HG on the gap junction function was further observed using the scrape-loading dye transfer technique (SLDT).^29^ The green fluorescence was widely distributed in the control group, but only concentrated at the scratch marks in the HG group. Although we detected the extracellular ATP content using the chemiluminescence method. The results showed that the extracellular ATP content was significantly increased in the HG group (Figure 4d). NPS R568 or Calhex231 pretreatment could reduce or increase the effect of HG (Figure 4c and d). Our results also showed that *β*-catenin (in cytoplasm and nucleus) and the p-GSK-3*β* were significantly increased in HG group, but the phosphorylation of *β*-catenin at Ser33 and Ser37 were decreased in HG group. NPS R568 or Calhex231 led to a similar effect as mentioned above (Figure 5).

### Activation of CaSR can inhibit the abnormal degradation of Mfn1, Mfn2 and Cx43 by attenuating ubiquitination

**Figure 6:**
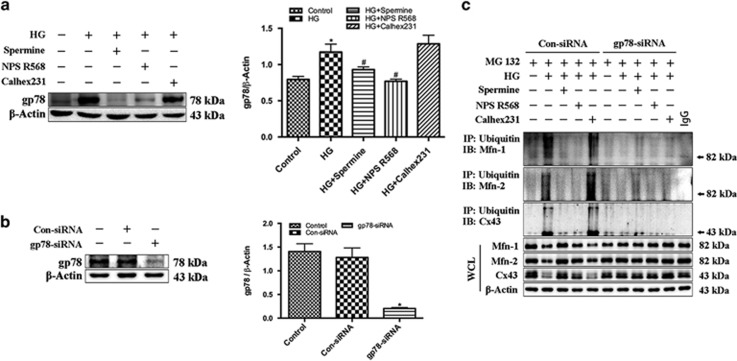
Activation of CaSR can attenuate the degradation of Mfn1, Mfn2 and Cx43 by HG-gp78-ubiquitin proteasome pathway. The expression of gp78 and the ubiquitination level of Mfn1, Mfn2, Cx43 were detected in cultured cardiomyocytes in control group (5.5 mM) and HG group (40 mM) after various treatment for 48 h. (**a**) Representative western blot of gp78 in comparison with *β*-actin expression in cardiomyocytes exposed to HG in the presence of 5 *μ*M NPS R568 or 3 *μ*M Calhex231 (*n*=3); (**b**) Representative western blot of gp78 in cardiomyocytes which were transfected with gp78-siRNA or Con-siRNA (*n*=3). (**c**) The ubiquitination level of Mfn1, Mfn2 and Cx43 in cardiomyocytes which were transfected with gp78-siRNA or Con-siRNA and treated by 5 *μ*M NPS R568 or 3 *μ*M Calhex231 respectively by immunoprecipitation (*n*=3). A representative blot is shown. *P<0.05 *versus* control; ^#^*P*<0.05 *versus* HG

We firstly detected the gp78 expression. The results showed that gp78 expression increased significantly in the HG group and Calhex231 group, while NPS R568 and Spermine obviously inhibited the action of HG (Figure 6a). These results suggested that activation of CaSR can inhibit gp78 expression.

We then utilized the gp78-siRNA to investigate the role of gp78-ubiquitin proteasome system in HG-induced cardiomyocyte injury. The results showed that the gp78-siRNA significantly inhibited the expression of gp78, but con-siRNA had little effect (Figure 6b). Compared with the control group, the ubiquitination levels of Mfn1, Mfn2 and Cx43 were markedly increased in HG group and Calhex231 group, which will inevitably decrease the Mfn1, Mfn2 and Cx43. CaSR agonists and gp78-siRNA can weaken the influence of HG (Figure 6c). Obviously, activated CaSR could protect cardiomyocytes by inhibiting the HG-induced activation of gp78-ubiquitin proteasome system.

## Discussion

In recent years, the morbidity and mortality of diabetes are increasing.^30, 31^ The major complication of diabetes is DCM,^32, 33^ which specific mechanism has not been clarified. In the previous study, we found that the cardiac contraction and relaxation in diabetic rats was decreased,^23^ which is related to the unbalanced calcium homeostasis caused by downregulation of CaSR expression. In present study, we, for the first time, explored that ATP flux disorder (the decrease of ATP synthesis and the increase of ATP leakage) is a crucial mechanism of DCM. We observed the effects of HG on ATP content, the expression of CaSR, mitochondria, gap junction and other related protein in primary cultured cardiomyocytes, and analyzed the role and mechanism of CaSR expression change in abnormal myocardial energy metabolism.

Myocardial contraction and relaxation both require a large amount of ATP, which is mainly generated in mitochondrial respiratory chain *in vivo*.^34^ Our results showed that both the CaSR expression and the ATP content of cardiomyocytes was significantly decreased in HG group, suggesting that the two effects may be associated. We also observed that the expression and activity of mitochondrial respiratory chain complex (*I*–*V*) related proteins were decreased in HG group, and the pretreatment of NPS R568 or Calhex231 could reduce or further aggravate the above changes. These results implicate the downregulated expression of CaSR is involved in the cardiac energy metabolism dysfunction induced by HG.

To explore the interaction of the downregulation of CaSR expression and the decreased expression and activity of mitochondrial respiratory chain complex (*I*–*V*) related proteins under HG treatment, the mitochondrial related structural proteins were further examined. The results showed that the expression of Fis1 and Drp1 were upregulated in HG group. By contrast, the expression of Mfn1 and Mfn2 were downregulated in HG group. Undoubtedly, the above-mentioned expression changes of Drp1, Fis1, Mfn2 and Mfn1 induced by HG will inevitably lead to the decrease of expression and activity of mitochondrial respiratory chain complex (*I*–*V*) protein, which certainly result in the decrease of ATP synthesis.

Mitochondria synthesize ATP by the oxidative phosphorylation and the electron transfer of mitochondrial respiratory chain.^24, 35, 36^ The later depends on the proton pump to maintain normal Δ*ψ*m.^37^ Thus, when the intracellular ATP content is reduced, the proton pump failure will inevitably lead to the decrease of Δ*ψ*m which will further reduce the production of ATP by affecting the electron transfer.^37, 38^ Therefore, the decrease of intracellular ATP content and Δ*ψ*m could form a vicious circle. Our present study observed that the mPTP was significantly open and the Δ*ψ*m was decreased in the HG group. NPS R568 or Calhex231 pretreatment can alleviate or aggravate the changes induced by HG. Obviously, the downregulation of CaSR expression is a critical mechanism leading to energy metabolism disorder induced by HG.

To analyze the reason for decrease of intracellular ATP content further, we simultaneously detected the extracellular ATP content (in cell culture medium). The results showed that the content of extracellular ATP was increased. As a water-soluble substance, ATP is difficult to pass the cell membrane. As reported, GJIC in the pathological state can form an open hemichannel, releasing ATP and other small molecules into the extracellular fluid.^39^ So, we observed the altered expression of connexin 43 (Cx43) and other related proteins in HG condition.

Connexin (Cx) is a basic protein of GJIC, which allows rapid transfer of inorganic ions, ATP and other small molecules between the cells.^12, 40^ Among the Cx superfamily members, the Cx43 is abundantly expressed in myocardium,^41^ and is critical to maintain the structure and function of cardiac gap junction. Clustered N-cadherin/*β*-catenin complexes at the plasma membrane appear to be a prerequisite for GJIC formed by Cx43.^28^ Furthermore, phosphorylation of Cx43 at Ser368 has been previously shown to cause GJIC dysfunction.^42^

Our experimental results showed that the expression of Cx43, N-cadherin and *β*-catenin protein were downregulated, the N-cadherin/*β*-catenin complexes were decreased, and p-Cx43 was significantly upregulated in HG group. CaSR agonists or inhibitor pretreatment can reduce or aggravate the above-mentioned changes. To further prove that the abnormal expression of Cx43 can cause the dysfunction of GJIC, SLDT was used. Our results showed that yellow lucifer (green fluorescence) were widely distributed among cells in control group, but only existed in scratch in HG group. NPS R568 pretreatment can lead the green fluorescence widely spread among cells in HG group.

These results collectively suggest that cardiac energy metabolism disorder caused by HG, not only related to the reduction of ATP synthesis induced by downregulating CaSR expression, but also with the increase of ATP leakage by dysfunction of GJIC.

The classical Wnt/*β*-catenin signaling pathway is involved in the early development of the embryo, organ formation, tissue regeneration and other physiological processes.^43, 44, 45^ Under pathological conditions, this pathway is also involved in the occurrence of cancer and metabolic syndrome.^46, 47^ We observed that the expression of *β*-catenin was downregulated on the cardiomyocytes membrane, but was upregulated in the cytoplasm and nucleus in HG group. At the same time, the expression of p-GSK-3*β* protein was increased, the p-*β*-catenin was decreased in HG group. NPS R568 or Calhex231 pretreatment can significantly inhibit or aggravate the above changes. The decreased expression of *β*-catenin protein on the cell membrane could inhibit the complex formation of N-cadherin and *β*-catenin, which will damage the integrity of the hemichannel,^48^ resulting in the increase of ATP leakage. Grol *et al.*^49^ found that increase of extracellular ATP could abnormally activate the Wnt/*β*-catenin pathway. Remarkably, the downregulation of CaSR expression have a crucial role in this process.

Why the activation of CaSR can reduce the adverse effects of HG on mitochondria and cell gap junction? We speculated that increase of intracellular calcium induced by activation of CaSR inhibit gp78 expression. As mentioned earlier, gp78 can degrade some corresponding proteins by ubiquitin proteasome pathway.^9, 50^ Present study found that the expression of gp78 was significantly upregulated, while the expression of Mfn1, Mfn2 and Cx43 were markedly downregulated in the HG group. CaSR agonist pretreatment could significantly reduce the changes above. Therefore, the activation of CaSR protect mitochondria and cell gap junction by inhibiting gp78-ubiquitin proteasome system.

To verify our speculation, we used siRNA to disrupt the synthesis of gp78 protein and detected the ubiquitination level of Mfn1, Mfn2 and Cx43 by immunoprecipitation. The results showed that the ubiquitination level of Mfn1, Mfn2 and Cx43 were significantly increased in HG group. The CaSR agonist and the gp78-siRNA both reduced the ubiquitination level of these proteins, and the CaSR inhibitor enhance the level of ubiquitination. Goetz JG *et al* reported that the expression of gp78 protein decreased with the increase of intracellular Ca^2+^.^10^ The activated CaSR can increase the intracellular Ca^2+^ by activating the G protein-PLC-IP3 pathway, which inhibit the abnormal protein ubiquitination induced by gp78.

**Figure 7:**
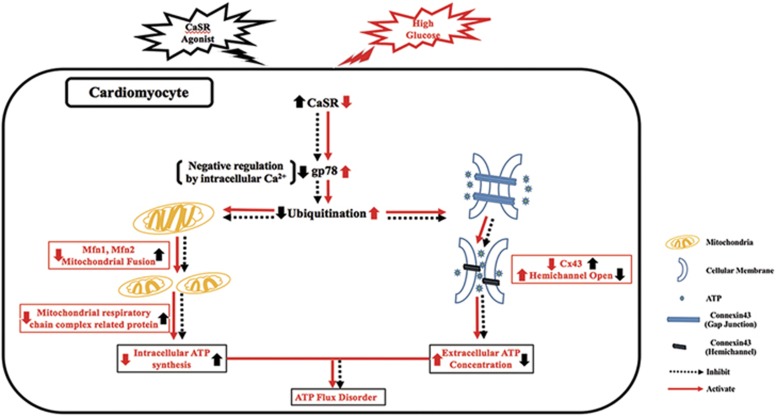
Schematic diagram on the mechanism of CaSR cardioprotection via attenuating high glucose-induced myocardial energy metabolism disorder. The downregulation of CaSR expression in cardiomyocytes induced by HG have a crucial role in myocardial energy metabolism disorder. The decreased CaSR expression causes upregulation of gp78 expression and activation of the ubiquitin proteasome system, which then degrades Mfn1, Mfn2, respiratory chain complex protein, Cx43, N-cadherin and *β*-catenin protein, and results in the decrease of ATP synthesis and the increase of ATP leakage through damaged gap junction. Therefore, CaSR agonists are expected to be a new target for the prevention and treatment of DCM

Based on the results and discussion of the present study, we suggest that HG induces downregulation of CaSR expression, which causes the activation of gp78-ubiquitin proteasome system. On the one hand, activated ubiquitin proteasome promotes the degradation of Mfn1 and Mfn2, resulting in the decrease of ATP synthesis. On the other hand, it degrades Cx43, causing ATP leakage (Figure 7). These changes destroy the ATP homeostasis (ATP flux). Therefore, the downregulation of CaSR expression is probably a crucial cause and mechanism of myocardial systolic and diastolic dysfunction in DCM.

## Materials and methods

### Isolation and culture of neonatal rat cardiomyocytes

Primary cultures of cardiomyocyte from neonatal Wistar rat aged 1–3 days were prepared, as previously described method.^51^ All the experiments were approved by the Animal Care Committee for the Use of Experimental Animals at Harbin Medical University (Heilongjiang, China). Briefly, in the super-clean worktable, the hearts were cut into pieces, digested with trypsin (Beyotime Biotechnology, Shanghai, China) for 8 min, then DMEM culture medium was added to terminate the digestion. After 8 times of the same process, the cells were collected by 10 min centrifugation with 600 *g* at 4 °C. Two hours after incubation at 37 °C, in a humidified atmosphere with 5% CO_2_, the attached cells were discarded and the unattached cells were continuously cultured. The cardiomyocytes were plated in collagen-coated 35 mm petri dish and maintained at 37 °C in a 5% CO_2_ humidified incubator in DMEM containing 10% fetal bovine serum (FBS) and 1% penicillin or streptomycin. The media was changed two times per week.

### Experimental protocols

The cultured cardiomyocytes were randomly divided into four groups: (1) Control group (Control): cardiomyocytes cultured with DMEM (glucose 5.5 mM, 10% FBS); (2) High glucose group (HG): cardiomyocytes cultured with DMEM containing high glucose (glucose 40.0 mM); (3) HG+NPS R568 group (HG+NPS R568): 5 *μ*M NPS R568 (Sigma-Aldrich, St. Louis, MO, USA) was added to the medium for 30 min before HG incubation. (4) HG+Calhex231 group (HG+Calhex231): 3 *μ*M Calhex231 (Sigma-Aldrich, St. Louis, MO, USA) was added to the medium for 30 min before HG incubation. The cardiomyocytes were incubated for 48 h.

### Mitochondria isolation

The mitochondria were isolated using previous method with slight modifications.^52, 53^ Briefly, cardiomyocytes were isolated by centrifugation and washed three times with PBS at 4 °C. Cells were resuspended with ice-cold hypotonic buffer (10 mM Tris–HCl, pH 7.6) containing protease and phosphatase inhibitor cocktails. The cell suspension was homogenized on ice using a glass homogenizer. The number of strokes was optimized by inspecting homogenate under the microscope for unintact cells. The cell homogenate was then gently passed twenty times through a 26 G 1/2 needle using a 1 ml syringe and centrifuged at 800 *g* for 10 min at 4 °C. The supernatant was collected in a 1.5 ml microcentrifuge tube and centrifuged again at 800 *g* for 10 min at 4 °C. The supernatant, which containing the crude mitochondria, was transferred to a new tube with 1.5 M sucrose solution which final concentration was 180 mM, then centrifuged at 14 000 *g* for 10 min to get the precipitation of mitochondrial. Finally, the specific lysis solution for mitochondria (Beyotime Biotechnology) was used to get the mitochondria related proteins.

### Isolation of cellular membrane

The cellular membrane was extracted by the Membrane and Cytosol Protein Extraction Kit (Beyotime Biotechnology). Briefly, Primary cardiac myocytes were inoculated into the 35 mm Petri dish. After drug treatment, the cells were scraped off by a cell scraper, centrifuged at 600 *g* for 5 min, put into a pre-cooled EP 1.5 ml tube with cell membrane extraction reagent A containing PMSF for 15 min. The cell suspension was homogenized on ice using a glass homogenizer. The number of strokes was 120 times. After centrifuged at 700 *g* for 10 min at 4 °C, the supernatant was carefully collected into the new 1.5 ml EP tube. To precipitate cell membrane fragments, 14000 *g* centrifugation for 30 min at 4 °C was used. The supernatant was discard and 200 *μ*l cell membrane extraction reagent B was added into this tube. After vortex shock 5 s and ice bath 10 min were repeated 4 times, the tube was centrifuged at 14000 *g* for 5 min, the cell membrane proteins in supernatant were collected.

### Cellular nucleus isolation

The Nuclear and Cytoplasmic Protein Extraction Kit (Beyotime Biotechnology) was used to isolate the cellular nucleus. The experimental procedure was carried out per the manufacturer’s protocol. The process is similar with the process for isolation of cellular membrane, except relevant reagent, centrifugal time and speed. Finally, the supernatant which containing the nuclear proteins was collected.

### Mitochondrial respiratory chain complex activity assay

Mitochondrial-enriched supernatants were prepared from neonatal rat primary cardiomyocytes samples, as previously described.^52, 53^ The activity of respiratory chain enzyme complexes I, II, III, IV and V (C-I, C-II, C-III, C-IV, C-V) in supernatants were assayed using the respiratory chain complex assay kit (GENMED, USA) and UV–VIS spectrophotometer (SHIMADZU, Japan) as the manufacturer’s protocol. For C-I, reaction time was 3 min. For C-IV, reaction time was 1 min. For C-II, C-III and C-V, reaction time was 5 min. All assays were performed in at least three times. The protein content of each sample was determined using a BCA assay (Beyotime Biotechnology). All activities were normalized to the total protein content.

### Determination of mitochondrial membrane potential

Δ*ψ*m was tested with a mitochondrial membrane potential assay kit (Santa Cruz, Bergheimer, Germany). Briefly, the cells soaked in JC-1 (1 ×) were incubated at 37 °C for 30 min, then were washed 3 times with phosphate buffered saline. The fluorescence intensity was measured with a fluorescent microscope (Olympus IX81, Olympus Corporation) at the excitation wavelength of 488 nm. Furthermore, JC-1 monomer emits the green fluorescence at 530 nm emission wavelengths and JC-1 aggregate emits the red fluorescence at 590 nm emission wavelengths. At last, the ratio of JC-1 (red/green) was calculated on pictures. The decline or rise of ratio represents the decrease or increase in Δ*ψ*m. All experiments were repeated three times independently.

### Assay of mPTP opening

Co-incubating Calcein-AM (Santa Cruz) and cobalt chloride (Sigma Chemical Co., St. Louis, MO, USA) was utilized to mensurate the changes of mPTP opening. Cardiomyocytes were plated in 35 mm petri dish (2 × 10^6^ cells/dish). After different treatments, the cells were stained with 2 *μ*M Calcein-AM in the presence of 5 mM cobalt chloride in the dark for 30 min at 37 °C. The fluorescence intensity was examined using a fluorescence microscope (Olympus IX81, Olympus Corporation) at 488 nm excitation and 525 nm emission wavelengths. Experiments were repeated three times independently.

### Mito-Tracker staining

Cardiomyocytes (1 × 10^5^) were cultured in a 35 mm petri dish. After treatments, the cells were stained with 300 nM Mito-Tracker Deep Red FM (Molecular Probes, Thermo Fisher Scientific, Eugene, OR, USA) for 20 min. Mitochondria were imaged using a fluorescence microscope (Olympus IX81, Olympus Corporation, Tokyo, Japan) and the average length of mitochondria was measured using Image J (National Institutes of Health, USA). Experiments were repeated three times independently.

### Measurement of ATP concentration

ATP concentration in primary cultured neonatal rat cardiomyocytes was measured using ATP Assay Kit (Beyotime Biotechnology) and the Luminoskan™ Ascent (Thermo Fisher Scientific), according to the instructions of manufacturer. The amount of ATP for each sample was determined using an ATP calibration curve and normalized expressed as pmol of ATP.

### Scrape-loading dye transfer

SLDT was utilized to evaluate the gap junction intercellular communication (GJIC) activity.29 Briefly, the cells of each group were grown in 35 mm petri dish and rinsed three times with PBS containing 0.01% Ca^2+^ and 0.01% Mg^2+^ (Ca^2+^, Mg^2+^- PBS). After that, 15 ml PBS containing 0.05% Lucifer yellow CH (Molecular Probes, Thermo Fisher Scientific) were added to cover the dish bottom and several cuts were made on the bottom of dish using a scalpel. The cells were incubated in the dye solution for 5 min then rinsed with PBS containing Ca^2+^ and Mg^2+^. Finally, the cells were fixed with 1 ml of 4% paraformaldehyde and photographed using a fluorescence microscope (Olympus IX81, Olympus Corporation). Experiments were repeated three times independently.

### Transfection of gp78-siRNA *in vitro*

Cardiomyocytes were seeded at equal number of cells (2.0 × 10^5^ per dish) in 35 mm petri dish and maintained in the absence of antibiotic culture medium for 24 h before transfection, then washed three times with PBS. Cardiomyocytes were transfected with Control siRNA (Con-siRNA) and gp78-siRNA (Santa, Dallas, TX, USA) using Lipofectamine™ 2000 transfection reagent from Invitrogen™ (Thermo Fisher Scientific, Scotland, UK). siRNA and the transfection reagent complex were added to the reduced serum media (Gibco™ Opti-MEM™, Thermo Fisher Scientific, UK) for 8 h, the transfection continued for another 24 h in serum-containing regular medium. After that, the cells were subjected to research.

### Immunoblotting

Cardiomyocytes were harvested and lysed in RIPA buffer containing PMSF. Proteins concentration was quantified using BCA Protein Assay Kit (Solarbio, Beijing, China). Equal amounts of proteins were boiled and then separated by sodium dodecyl sulfate polyacrylamide gel electrophoresis (SDS-PAGE). The separated proteins were electrophoretically transferred into an aperture of 0.45 *μ*m polyvinylidene fluoride membrane (Merck Millipore, Massachusetts, USA). Membranes were blocked using Tris-Buffer Saline containing 5% non-fat milk (Becton, Dickinson and Company, New Jersey, USA) for 1 h at room temperature. Thereafter, they were incubated overnight at 4°C along with specific primary antibodies. CaSR, Mfn1, Mfn2, Cx43, P-Cx43 were purchased from Santa (Dallas, TX, USA); Drp1, *β*-catenin, p-*β*-catenin, N-cadherin, GSK-3*β*, p-GSK-3*β* antibodies were purchased from Cell Signaling Technology (Danvers, MA, USA); gp78, Hsp70, UQCRQ, ND1, ATP5F1, COX5A, SDHA, *β*-actin were purchased from Proteintech (Wuhan, China). The membrane was washed three times with 1 × Tris-Buffer Saline-Tween 20 (TBST) buffer and then incubated in TBST solution with horseradish peroxidase-labeled secondary antibody for 2 h at room temperature. Finally, the membrane was washed thrice with TBST solution. The signals were detected by the Enhanced Chemiluminescent (ECL) kit (HaiGene, Harbin, China) and the Multiplex Fluorescent Imaging System (ProteinSimple, California, USA). VDAC was used to confirm equal mitochondria loading. Na^+^-K^+^-ATPase was used as an equal cellular membrane control and Histone 3 as an equal nucleus loading. *β*-actin was employed to confirm equal cytosol loading. The intensities of protein bands were quantified by a Bio-Rad ChemiDoc™ EQ densitometer and Bio-Rad Quantity One software (Bio-Rad Laboratories, Hercules, CA, USA).

### Immunoprecipitation

Cardiomyocytes were seeded in 35 mm petri dish. After treatments, cells were collected and lysed in lysis buffer plus PMSF (Roche) for 30 min at 4 °C. After 14000 g centrifugation for 20 min, the lysates were immunoprecipitated with 2 *μ*g specific antibody of anti-N-cadherin (Cell Signaling Technology) overnight at 4 °C before coupled to Protein A/G Magnetic Beads (Selleckchem, Houston, TX, USA) for 2 h according to the instructions of Protein A/G Magnetic Beads for IP (Biotool). After that, SDS-PAGE and other protocal were performed using the above methods of immunoblotting.

### *In vitro* ubiquitylation assay

After transfection, cardiomyocytes were treated with 10 *μ*M MG132 (proteasome inhibitor, Selleckchem) for 6 h before collection. Then they were harvested and lysed in 1% SDS buffer (Tris pH 7.5, 0.5 mM EDTA, 1 mM DTT). The samples undergone the pull-down assays using anti-ubiquitin antibody and the precipitates were analyzed by immunoblotting using anti-Mfn1, anti-Mfn2 and anti-Cx43.

### Statistical analysis

All experiments were replicated at least three times independently. Differences between groups were analysed using one-way analysis of variance (ANOVA). The results are presented as the mean±standard error. *P*<0.05 were considered statistically significant.
