# Correction: Calcium sensing receptor protects high glucose-induced energy metabolism disorder via blocking gp78-ubiquitin proteasome pathway

**DOI:** 10.1038/s41419-020-02957-1

**Published:** 2020-09-21

**Authors:** Yuehong Wang, Ping Gao, Can Wei, Hongzhu Li, Li Zhang, Yajun Zhao, Bo Wu, Ye Tian, Weihua Zhang, Lingyun Wu, Rui Wang, Changqing Xu

**Affiliations:** 1 Department of Pathophysiology, Harbin Medical University, Harbin, 150086 China; 2 The Key Laboratory of Cardiovascular Medicine Research (Harbin Medical University), Ministry of Education, Harbin, 150086 China; 3 Department of Endocrinology, The Second Affiliated Hospital of Harbin Medical University, Harbin, 150086 China; 4 The Cardiovascular and Metabolic Research Unit, Laurentian University, Sudbury, ON Canada P3E 2C6; 5 Health Sciences North Research Institute, Sudbury, ON Canada P3E 5J1

Correction to: *Cell Death & Disease*

10.1038/cddis.2017.193 published online 18 May 2017

Since online publication of this article, the authors noticed that there was an error in Figs. 2 and 4. In Fig. 2c the samples were mislabelled, the correct labelling order is ‘Control, HG, HG + NPS R568, HG + Calhex231’. In Fig. 4c, an incorrect image was used to compile the HG + NPS R568 group, meaning the control was accidentally duplicated. The corrected images are provided below. The authors confirm that these errors did not influence the reported data, discussion, or conclusion. The authors apologise for any inconvenience to readers arising from this error.

**Figure:**
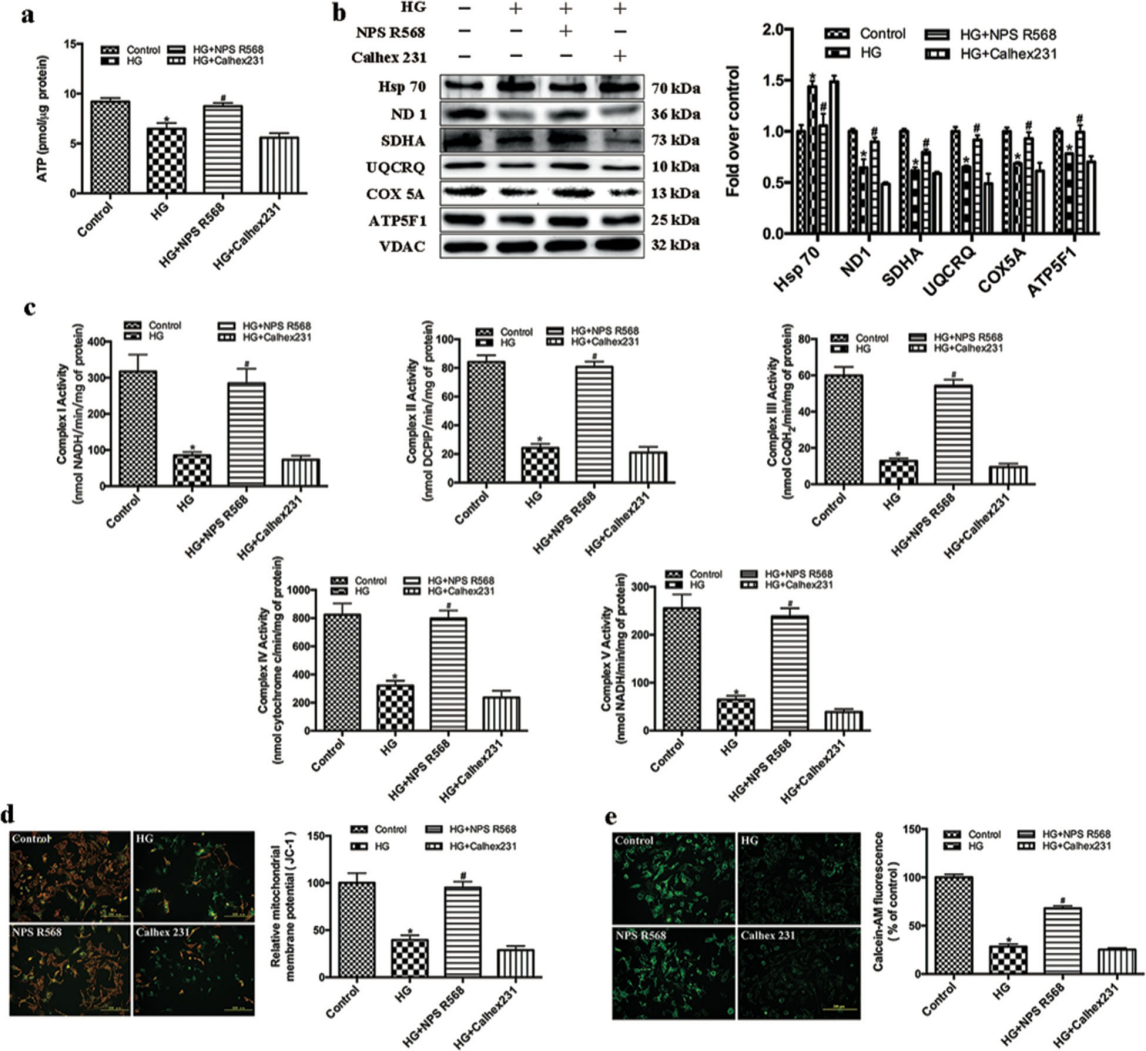
Fig. 2.

**Figure:**
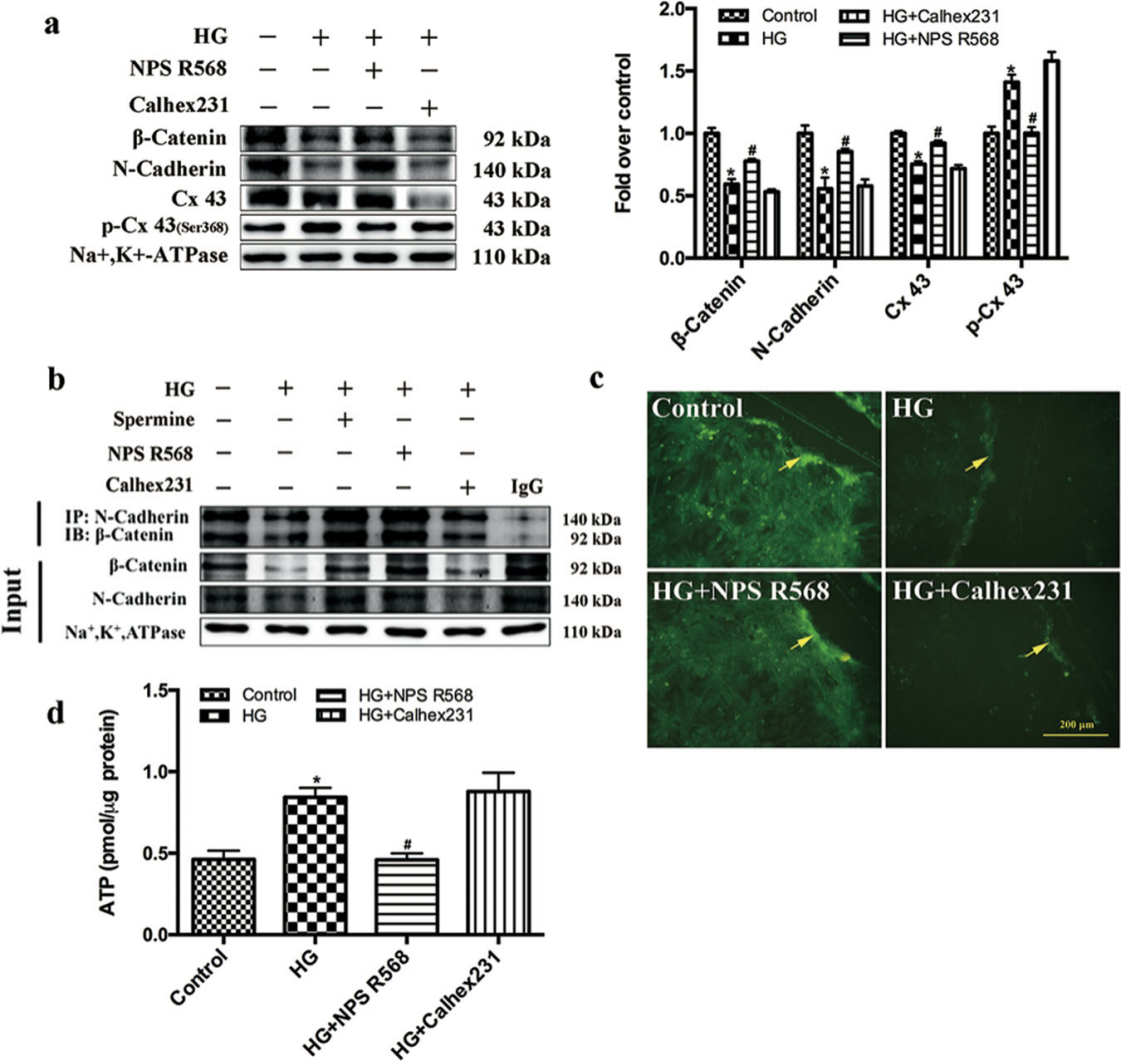
Fig. 4.

